# Light-absorption-driven photocatalysis and antimicrobial potential of PVP-capped zinc oxide nanoparticles

**DOI:** 10.1038/s41598-023-41103-7

**Published:** 2023-08-24

**Authors:** Karanpal Singh, Nancy, Monika Bhattu, Gurjinder Singh, Nabisab Mujawar Mubarak, Jagpreet Singh

**Affiliations:** 1 Department of Electronics Engineering, Sri Guru Granth Sahib World University, Fatehgarh Sahib, Punjab 140406 India; 2 Department of Biotechnology, Sri Guru Granth Sahib World University, Fatehgarh Sahib, Punjab 140406 India; 3 Department of Chemistry, University Centre for Research and Development, Chandigarh University, Gharuan, Mohali, 140413 India; 4 Department of Electrical and Electronics & Communication Engineering, DIT University, Dehradun, Uttarakhand 248009 India; 5 Petroleum and Chemical Engineering, Faculty of Engineering, Universiti Teknologi Brunei, Bandar Seri Begawan, BE1410 Brunei Darussalam; 6 Department of Biosciences, Saveetha School of Engineering, Saveetha Institute of Medical and Technical Sciences, Chennai, India

**Keywords:** Environmental social sciences, Materials science, Nanoscience and technology

## Abstract

Toxic dyes in water bodies and bacterial pathogens pose serious global challenges to human health and the environment. Zinc oxide nanoparticles (ZnO NPs) demonstrate remarkable photocatalytic and antibacterial potency against reactive dyes and bacterial strains. In this work, PVP-ZnO NPs have been prepared via the co-precipitation method using polyvinylpyrrolidone (PVP) as a surfactant. The NPs’ microstructure and morphology were studied using X-ray diffraction (XRD), having a size of 22.13 nm. High-resolution transmission electron microscope (HR-TEM) and field emission scanning electron microscopy (FESEM) analysis showed spherical-shaped PVP-ZnO NPs with sizer ranging from 20 to 30 nm. Fourier Transform Infrared Spectroscopy (FT-IR) confirmed the hybrid nature of the NPs, and UV–Vis spectroscopy showed an absorption peak at 367 nm. The PVP-ZnO NPs exhibited high photocatalytic activity, achieving 88% and nearly 95% degradation of reactive red-141 azo dye with 10 mg and 20 mg catalyst dosages, respectively. The antibacterial properties of the NPs were demonstrated against *Escherichia coli* and *Bacillus subtilis*, with inhibition zones of 24 mm and 20 mm, respectively. These findings suggest that PVP-ZnO NPs can be effectively used for water treatment, targeting both dye and pathogenic contaminants.

## Introduction

The textile industry has recently drawn much attention due to numerous dyes posing poisonous and cancer-causing endangering effects on all tropical species. Additionally, the dyes containing waste products from other businesses that produce food, process leather, make paper, print, use paints, and cosmetics pose serious environmental hazards due to their leakage into freshwaters. Among all the dye effluents, azo dyes are the most popular and are quite dangerous. They are complex aromatic molecules, and in general, the structure of the dye is stable^1–4^. Because their limited degradability and incomplete degradation of the dye led to numerous toxic compounds, hence are resistant to wastewater handling techniques8. Among the various azo dyes, reactive red-141 azo dye is widely used in textile industries and has been reported as the most dangerous to the environment and human health. Therefore, the immediate removal of these dyes from natural water sources has become important.

Adsorption, membrane separation, physical and chemical coagulation, advanced oxidation processes (AOPs), and biodegradation are some methods reported for wastewater treatment^5^. However, due to the advantages of minimal energy input, simple operation, cleanness, and efficiency, semiconductor photocatalysis technology has been widely used to degrade pollutants and decompose water to produce hydrogen^6,7^. Photocatalytic oxidation has garnered significant attention in environmental protection research, primarily for its utility in the photodegradation of organic pollutants. This technology boosts several notable advantages, including cost-effectiveness in terms of operational expenses, exceptional efficacy in removing intricate chemical compounds, absence of reliance on supplementary materials, harnessing of freely available solar energy, and the ability to execute the process under ambient temperature and pressure conditions. Extensive research efforts have led to the development and utilization of numerous nanomaterials fabricated from semiconducting oxides and sulphides, effectively applied in the photocatalytic application^8,9^.

Moreover, the emergence of bacterial pathogens in water bodies is also a worldwide issue. Recently, bacterial pathogens have been consistently identified in wastewater treatment plants, suggesting that these plants serve as significant reservoirs for the proliferation of various pathogenic microorganisms. Numerous molecular techniques have been documented in scientific literature to identify bacterial species inside environmental samples^10–13^.

Numerous scientific investigations have been conducted to delve deeper into the antibacterial properties of nanoparticles, revealing a multitude of mechanisms by which they effectively inhibit the growth and survival of microbes. Metal nanoparticles, including silver, gold, zinc and magnesium, have been found to exhibit potent antibacterial capabilities. In addition to metal nanoparticles, it has been documented that metal oxide nanoparticles exhibit antibacterial properties^14^. Capitalizing on metallic nanoparticles is a promising approach to combat these bacterial strains, encompassing both gram-positive and gram-negative classifications. Inorganic antibacterial compounds have recently been employed to control organisms in various industries^15,16^. Inorganic metal oxide bulk materials are reduced to the nanoscale, which changes their activity and improves their physical, chemical, and biological characteristics^17,18^. Highly reactive metal oxide nanoparticles have effectively killed gram-positive and gram-negative bacteria.

Nanoscale metal oxide semiconductors are the most spectacular, well-known, and in-demand materials in various fields due to their size, shape, reactivity, and optical and electrical properties. These nanomaterials’ piezo electronic, optoelectronic, and catalytic capabilities make them suitable for solar cells, biosensors, light-emitting diodes, transistors, and light-emitting diodes^19,20^. The degradation of organic compounds is one of these applications where heterogeneous photocatalysis excels and triumphs. Due to its biocompatibility and excellent photostability, ZnO is an excellent photocatalyst prospect^21^. Its outstanding physical and chemical qualities, such as its high chemical stability, electrochemical coupling coefficient, and broad light absorption range, make it a material with many uses. It is an innovative and versatile n-type semiconducting material, and its electrons can act as charge carriers to transition from the valence band to the conduction band. A direct large band gap of ZnO is to be 3.37 eV^22^. Considering where Zn and O are on the periodic table, it is sometimes called an II–VI semiconductor. There is considerable interest in using nanoparticles made of metals and their oxides. Zinc (Zn) and its oxide are among the well-researched metals that impact living items (ZnO). Owing to its powerful reduction characteristics, zinc is an active element. Zinc oxide may be produced readily by oxidation. ZnO is an inorganic semiconductor material with three distinct crystal structures—wurtzite, zinc blende, and rock salt^23,24^. The structure of wurtzite, in which each zinc atom is tetrahedrally coupled with four oxygen atoms, is thermodynamically stable under ambient circumstances. Furthermore, ZnO nanoparticles offer several benefits, including action against different bacteria, and play an important role in cleaning polluted industrial water using a low dose of nanoparticles.

In line with the above discussion, the present investigation employs co-precipitation to synthesize zinc oxide nanoparticles (PVP-ZnO NPs). The synthesized PVP-ZnO NPs are subsequently employed for photocatalytic and antimicrobial purposes. This exploration of PVP-ZnO NPs imparts a novel understanding of the interrelationship between their inherent properties and potential applications. Despite numerous studies on the synthesis and utilization of zinc oxide nanoparticles (PVP-ZnO NPs) as photocatalysts and antimicrobial agents, the present investigation delves into the evaluation of the underlying mechanisms of reactive oxygen species (ROS) and 2,2-diphenyl-1-picrylhydrazyl (DPPH) assays. This approach contributes an extensive and comprehensive analysis, providing a more profound understanding of the subject matter.

## Materials and methods

### Chemicals used during the synthesis process

The specific chemicals utilized, namely Polyvinylpyrrolidone (C_6_H_9_NO)n, Zinc Nitrate Hexahydrate, and Sodium Hydroxide (NaOH), were acquired from Sigma-Aldrich, Germany. De-ionized (DI) water was consistently employed throughout the experimental process.

### Synthesis process

In this study, 0.64 g of zinc nitrate was thoroughly dissolved in 25 mL of deionized water and then continuously agitated for 15 min at room temperature at 200 revolutions per minute (rpm). Polyvinylpyrrolidone (PVP) solution was prepared individually using 10 mL DI water and agitated at ambient conditions. The zinc solution was then added gradually to the prepared PVP solution, and the reaction mixture was kept at 70 °C for 2–3 h while stirred at 350 rpm. Meanwhile, NaOH solution_*aq.*_ (2.05 g in 40 mL DI) was added dropwise to the resulting liquid to maintain a pH of 9.1. Addition of base to the reaction mixture results in the formation of milky white precipitates. After two to three hours of heating and stirring, the solution was left at room temperature to cool and let the sediments settle. The solution was centrifugated at a speed of 5000 rpm for 4 min, facilitating the collection of precipitates in a centrifuge tube. Subsequently, the precipitates were washed with DI water to eliminate undesired elements. Finally, the gathered precipitates were placed and dried in a hot oven for 24 h at 60 °C. The acquired white color powder was ground into a fine powder in mortar and pestle to create PVP-ZnO NPs. This paper discusses a low-temperature, low-cost, and high-yield (> 85%) method for synthesizing ZnO nanostructures^25,26^.

$${\text{Zn}} {({\text{NO}}}_{3}{)}_{2}+\mathrm{ PVP }+ 2\mathrm{NaOH }\to {\text{Zn}} {({\text{OH}})}_{2} + 2{\text{NaNO}}_{3},$$

$${\text{Zn}} {({\text{OH}})}_{2}\to {\text{ZnO}}+{\text{H}}_{2}{\text{O}}.$$

### Characterization techniques

The crystallographic properties of the synthesized PVP-PVP-ZnO NPs were characterized using a Panalytical X'PERT PRO X-ray diffractometer (XRD) from the Netherlands, employing Cu Kα (1.5406 Å) monochromatic radiation. The Shimadzu-1800 UV–Vis spectrophotometer was used to measure the absorption spectra over the wavelength range of 200–800 nm. The chemical bonding of the materials was determined by analyzing the transmittance spectra between 4000 and 600 cm^−1^ through a Bruker FT-IR Spectrometer. Germany-based Carl Zeiss Model Supra 55 The surface morphological properties of the NPs were examined using field emission scanning electron microscopy (FESEM) in conjunction with EDX for elemental content percentages. PHI 5000 VersaProbe III by Physical Electronics was used to measure XPS data. The morphological, physical characteristics and grain size of the NPs were investigated using a Thermo Fisher TALOS F200 S High-Resolution Transmission Electron Microscope (HRTEM) working at 200 kV. The particles were dispersed in ethanol via ultrasonic treatment to facilitate the sample preparation. Subsequently, the resulting suspension was deposited onto a carbon-coated copper grid and dried before capturing the HRTEM images.

### Assessment of antibacterial potency

Two strains, *Escherichia coli* (ATCC25922) and *Bacillus subtilis* (DSM6633), were employed to assess the antibacterial effectiveness of the synthesized samples. These bacterial strains were sourced from the biotechnology laboratory of Sri Guru Granth Sahib World University. These strains were incubated at 37 °C overnight while sub-culturing on Luria Bertani medium (LB). To evaluate the antibacterial activity, the agar diffusion method was used. The homogeneous inoculation of the LB medium was carried out using a sterile swab of bacterial strain (Luria–Bertani). Subsequently, LB-agar plates were punctured with 3–4 wells to accommodate the prepared samples, available in four different concentrations: 100 μg/mL, 600 μg/mL, 1 mg/mL, and 3 mg/mL. Following incubation at 37 °C for 24 h, the diameter of the inhibitory zones surrounding the wells was measured. The zone of inhibition was measured from the well's centre outward to the point where bacterial growth first appeared. For various concentrations, distilled water was used to dissolve each sample.

### Free-radical scavenging activity

The DPPH test assessed the free radical scavenging activity of PVP-ZnO NPs, as previously described in the literature^27,28^. This activity used 2,2 diphenyl-1-picrylhydrazyl, free radical DPPH, and methanol. The DPPH solution was made in the dark using magnetic stirring for 30 min. In absolute methanol, a 15 mM concentration of DPPH solution was made. 100 μL of PVP-ZnO NPs with concentrations of 10, 20, 30, and 40 µg/mL were added to 200 µL of DPPH solution for this test. The mixture was briskly agitated and allowed to stand for 1 h. After 1 h, the absorbance at 518 nm was measured to determine the decrease of the DPPH radical. As a positive control, ascorbic acid was used. The RSA was calculated by using the equation:

$$DPPH Scavenging= \frac{{A}_{o}-A}{{A}_{o}}\times 100,$$

where A_0_ and A are the absorbances of (DPPH·) in solutions.

### Statistical analysis

The experiments were performed three times. Mean ± Standard Deviation (SD) is used to represent the values. The Origin 9.0 software computed the ZnO inhibition value.

### Photoactivity activity

The degradation rate of reactive red 141 was used to evaluate the photocatalytic activities of nanoparticles under solar light. In a working experiment, 5 mg/L of RR 141 solution was mixed with 20 mg/L of catalyst (PVP-ZnO NPs) with constant stirring. Then, sunlight was used to illuminate the solution. Approximately 5 mL of sample was collected after photo-irradiation, centrifuged to obtain the supernatant, and then analyzed using the UV–Vis spectrophotometer.

## Results and discussions

### XRD analysis

X-ray diffraction (XRD) analysis determines the unit cell’s phase composition and lattice spacings (d-values) in the crystalline material. This information is crucial for determining the crystal structure and crystallite size of the synthesized PVP-ZnO NPs. A crystalline material’s X-ray diffraction (XRD) pattern represents a unique fingerprint characterized by multiple diffraction peaks with varying intensities. This collected XRD data is then compared to readily available reference databases to identify the phases present in the material.

**Figure 1:**
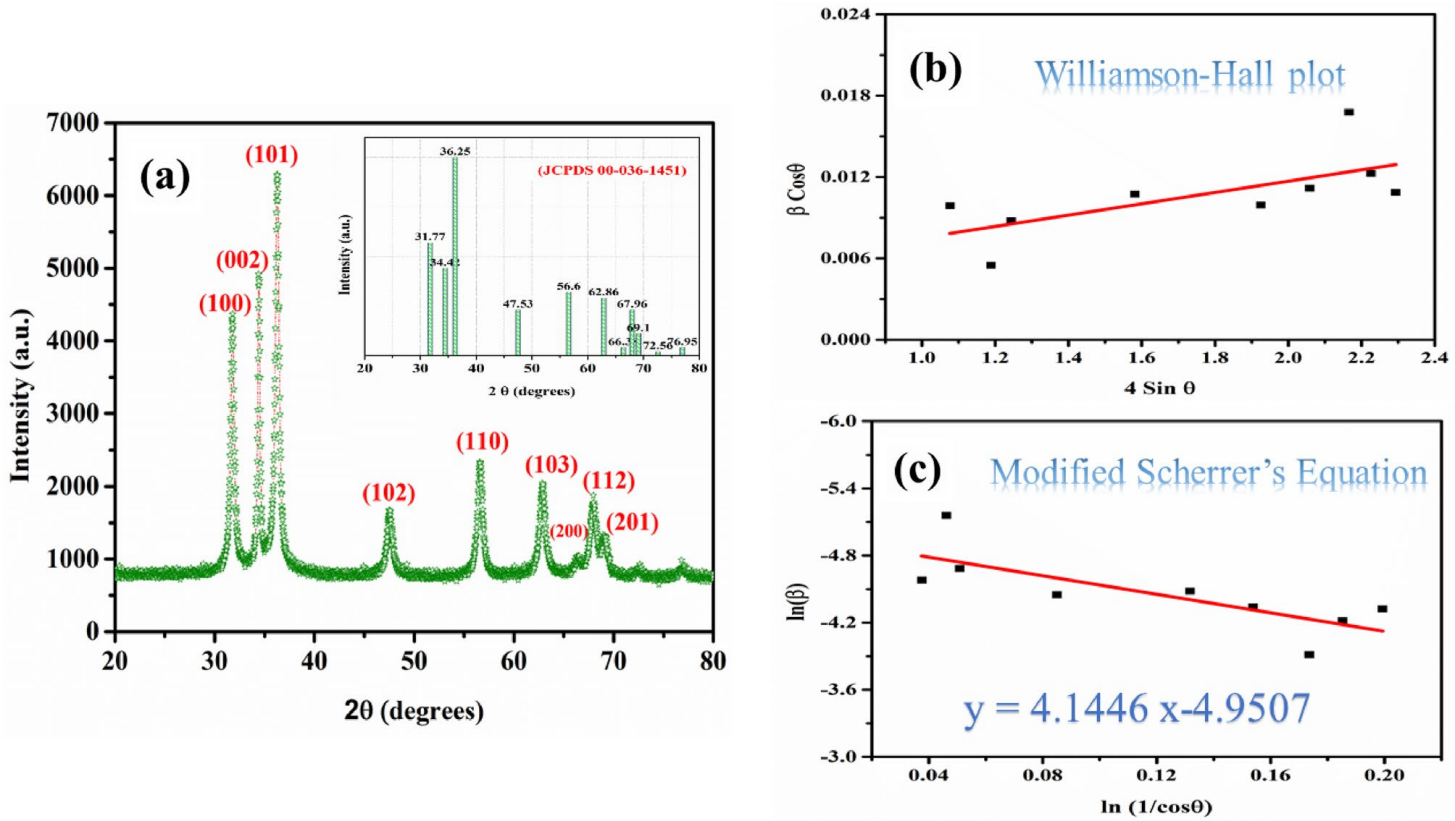
(**a**) XRD spectrum of Chemical synthesized ZnO Nanoparticles along with JCPDS ruler (inset), (**b**) Williamson–Hall plot, and (**c**) modified Scherrer’s Equation.

Figure 1 depicted the XRD pattern of PVP-ZnO NPs and the peaks at 2θ = 31.2268°, 34.5679°, 36.2348°, 46.5897°, 57.5491°, 61.9624°, 65.5796°, 67.6246°, and 69.9730° correspond to (100), (002), (101), (102), (110), (103), (200), (112), (201) diffraction planes, respectively as shown in Table 1. All diffraction peaks can be indexed by the hexagonal wurtzite-type structure, which is in accordance with JCPDS No. 00-036-1451^29^. The crystalline parameters of the unit cell provided by XRD and the XRD pattern matched with standard JCPDS data confirm its wurtzite hexagonal structure. An outstanding planar similarity was attained when measured against the generated PVP-ZnO NPs. By excluding any other reflections in the pattern that are either not ZnO or associated with contaminants, it is shown that the spherical shape particles are pure wurtzite hexagonal phase ZnO. The peaks were evidence of the consistent hexagonal structure of wurtzite. From the data, particle size was calculated using the Debye Scherrer equation, which was around 22.13 nm. The crystal size is determined from the above data using Scherrer Debye’s Eq. (1)^30^.

1 $$D=\frac{k\lambda }{\beta {\text{cos}}\theta },$$

where k = 0.95–0.98 (shape factor), λ = 0.154 nm (X-ray wavelength), β = half width of the diffraction band FWHM (radians), θ = Bragg’s diffraction angle.

**Table 1:** Crystalline parameters of synthesized PVP-ZnO NPs.

Sr no.	2 Ɵ (degree)	FWHM	Lattice planes	Inter-planner spacing (d)	The crystallite size (D) nm
1.	31.2268	0.58745	100	2.7562	24.56985
2.	34.5679	0.32964	002	2.8561	37.87965
3.	36.2348	0.52865	101	2.3964	21.2651
4.	46.5897	0.66961	102	1.7531	20.36579
5.	57.5491	0.64872	110	1.4452	19.85460
6.	61.9624	0.74612	103	1.3678	18.6941
7.	65.5796	1.14328	200	1.4539	19.6951
8.	67.6246	0.84569	112	1.3149	17.3248
9.	69.9730	0.75981	201	1.3015	19.6218
					D (avg.) = 22.13

The calculated size by using Scherrer’s equation is = 22.13 nm.

2 $${\beta }_{\tau }{\text{cos}}\theta =\varepsilon \left(4{\text{sin}}\theta \right)+k\lambda /D,$$

where β = Total broadening, θ = Bragg’s angle, k = 0.95–0.98 (shape factor), λ = 0.154 nm (X-Ray wavelength).

The calculated size by using the Williamson-Hall equation is = 28.61 nm, as shown in Table 2.

3 $${\text{ln}} \left(\beta \right)={\text{ln}}\frac{1}{{\text{cos}}\theta }+\frac{{\text{ln}}k\lambda }{D},$$

where ln = taking log on both sides, k = 0.95–0.98 (shape factor), λ = 0.154 nm (X-ray wavelength) β = half width of the diffraction band FWHM (radians), θ = Bragg’s diffraction angle.

**Table 2:** Results of calculations using the Williamson–Hall equation.

Sr no	2θ degrees	Theta (radians)	FWHM (degrees)	FWHM (radians)	β cos θ	4 sin θ
1	31.2268	0.272505237	0.58745	0.010252937	0.009875	1.07658
2	34.5679	0.301661835	0.32964	0.005753303	0.005494	1.18843
3	36.2348	0.316208282	0.52865	0.009226683	0.008769	1.24386
4	46.5897	0.406571831	0.66961	0.011686899	0.010734	1.581852
5	57.5491	0.502210638	0.64872	0.0113223	0.009924	1.925458
6	61.9624	0.540723946	0.74612	0.013022251	0.011164	2.059027
7	65.5796	0.572289971	1.14328	0.019954	0.016775	2.166234
8	67.6246	0.590135963	0.84569	0.014760075	0.012264	2.225896
9	69.973	0.610629619	0.75981	0.013261186	0.010865	2.293534

Using a modified form of Scherrer’s equation, the size is calculated to be = 21.87 nm, as depicted in Table 3.

**Table 3:** Results of calculations using the modified Scherrer equation.

Sr no.	Peak position	FWHM	ln(1/cos θ)	ln(β)
1	31.2268	0.58745	0.037598397	− 4.580191108
2	34.5679	0.32964	0.046207235	− 5.157981094
3	36.2348	0.52865	0.050849878	− 4.685655657
4	46.5897	0.66961	0.085033047	− 4.449286791
5	57.5491	0.64872	0.131795093	− 4.480981053
6	61.9624	0.74612	0.15392508	− 4.341095799
7	65.5796	1.14328	0.173564381	− 3.914325641
8	67.6246	0.84569	0.18528792	− 4.215829382
9	69.973	0.75981	0.199320625	− 4.322913842

### UV–Visible spectroscopic analysis

**Figure 2:**
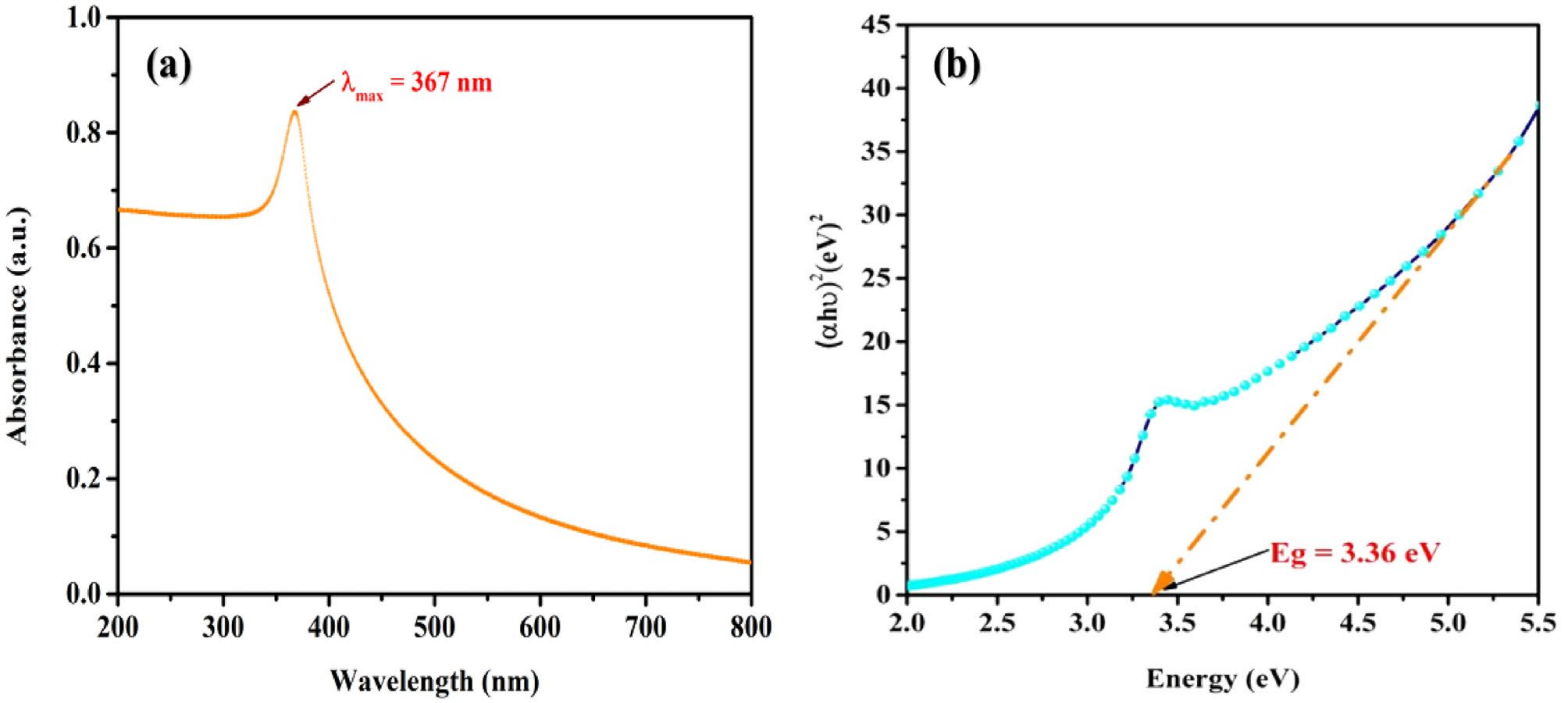
(**a**) UV–Vis spectrum, (**b**) Tauc’s plot of chemically synthesized PVP-ZnO NPs.

At room temperature, UV–Vis spectroscopy was used to examine the optical properties of the freshly manufactured spherical-shaped ZnO nanomaterial. The results are shown in Fig. 2a. For UV–Vis measurements, ZnO spheres (powder form) are well-dispersed in water, and the solution of dispersed ZnO water was used to track absorption over the wavelength range of 200–800 nm. As can be seen, the resulting UV–Vis spectrum has a prominent excitonic absorption peak at 367 nm, which corresponds to bulk ZnO in the hexagonal phase of wurtzite. The excitonic absorption peak at 367 nm is the only peak seen in the spectrum, demonstrating the ZnO nanostructures’ purity and exceptional optical properties.

A Tauc’s plot of the UV–Visible spectrum was plotted, and the related energy bandgap was identified in Fig. 2b. According to this spectrum, direct E_g_ transitions in synthetic ZnO Nano spherical should have an energy of roughly 3.36 eV, nearly equal to bulk zinc oxide. Using Eq. (2), the direct band gap energy was calculated^31,32^.

4 $${(\alpha h\nu )}^{2 }=A\left(h\nu -Eg\right){ }^{n},$$

where h = Planck’s constant, $\nu$ = frequency of vibration, $\alpha$ = absorption coefficient, Eg = band gap, A = proportional constant.

### FTIR analysis

**Figure 3:**
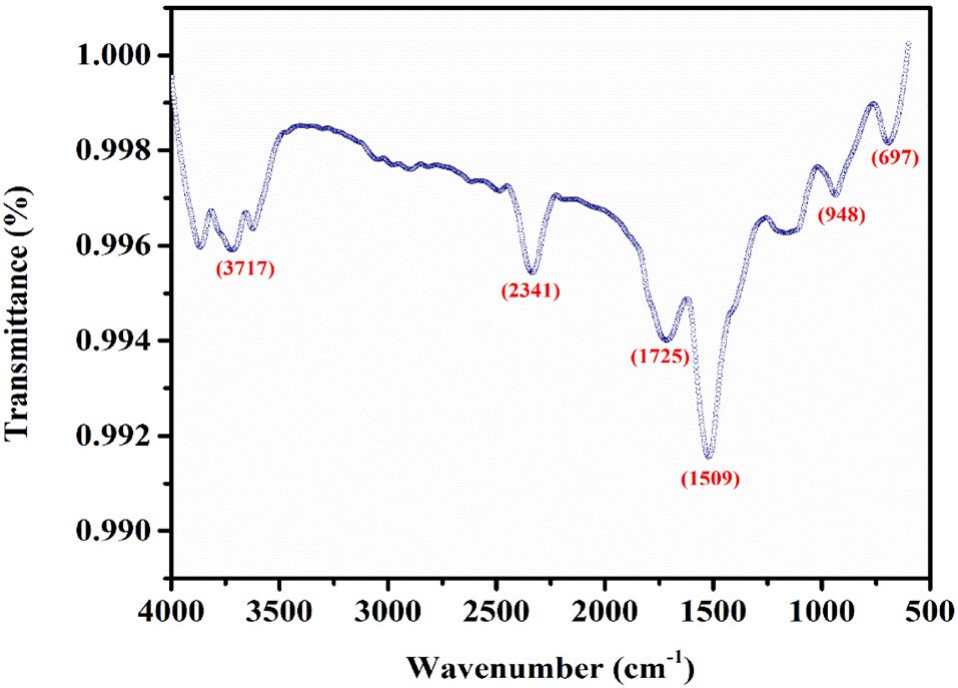
FTIR spectrum of chemical synthesized PVP-ZnO NPs.

In Fig. 3, FTIR analysis of PVP-ZnO NPs is shown. The transmittance band at 697 cm^−1^ for PVP-ZnO NPs may be attributed to Zn–O stretching, proving that the PVP-ZnO NPs are positively generated. The presence of water adsorption on the surface of the nanoparticles is evident from the observed bands at 3717 cm^−1^, which correspond to the stretching vibrations of the hydroxyl (O–H) groups. The band observed at 2341 cm^−1^ can be attributed to CO_2_ in the ambient air. Additionally, the stretching vibration of the C=O bond is observed at 948 cm^-1^, and the C–H vibration mode is shown on the band at 1725 cm^−1^^33,34^. Table 4 shows the FTIR spectra peaks.

**Table 4:** FTIR data with different modes.

S.no.	PVP-ZnO NPs (cm^−1^)	Modes
1.	697	Zn–O
2.	948	C=O (asymmetric stretching)
3.	1059	CH_2_OH (carbohydrate group)
4.	1509	Absorption peak
5.	1725	C–H
6.	2341	CO_2_
7.	3673	O–H (hydroxyl group)
8.	3717	
9.	3823	

### FESEM analysis

**Figure 4:**
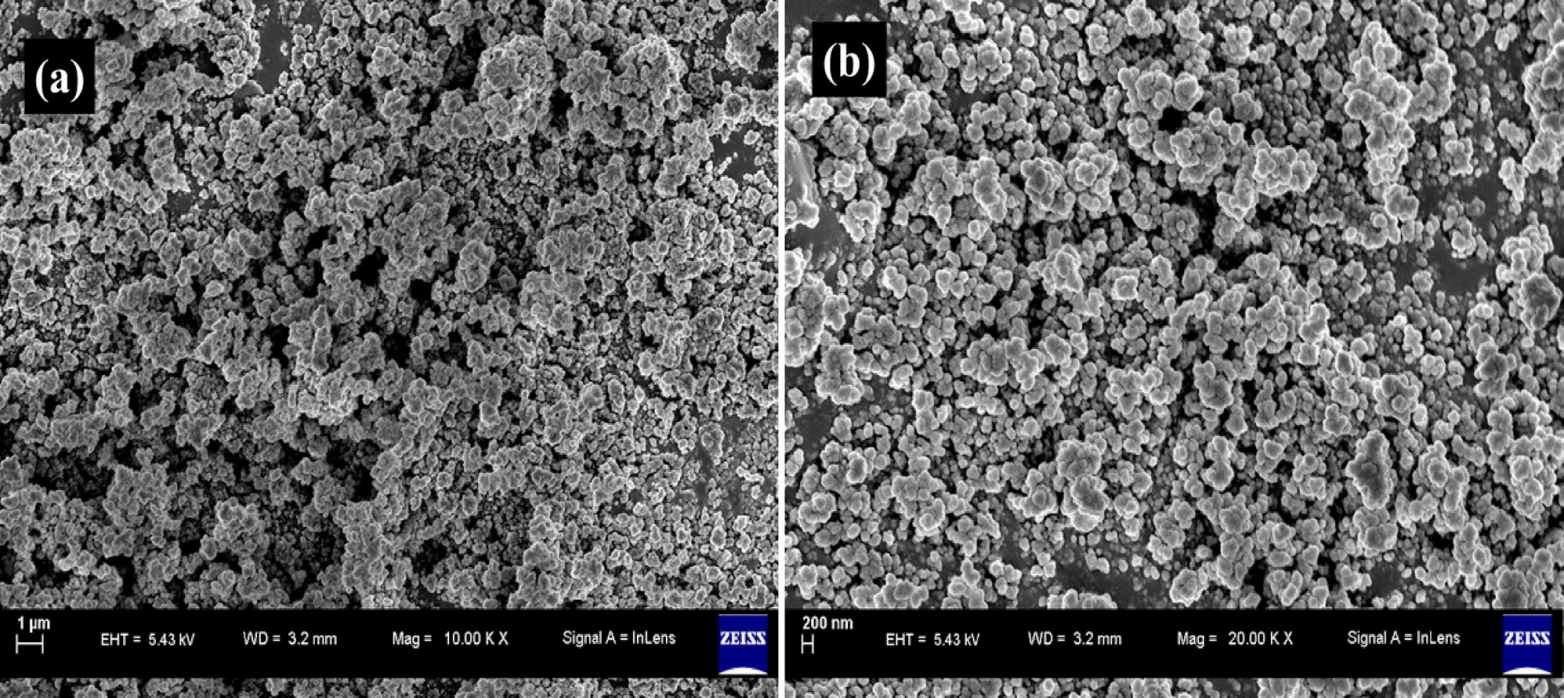
FESEM images of chemically obtained PVP-ZnO NPs (**a**) low magnification at 1 μm, (**b**) high magnification at 200 nm.

The chemically prepared PVP–PVP-ZnO NPs morphology was further examined using field emission scanning electron microscopy (FESEM). The analysis confirmed that the NPs have a spherical morphology, as shown in Fig. 4. Additionally, the FESEM images revealed that the particles are ordered into nanostructures and tend to clump together. The agglomeration of NPs is caused by the feeble adhesion of particles to one another, resulting in (sub)micron-sized entities. In contrast, NPs aggregates result from forming covalent or metallic bonds that are difficult to break. The spherical form of the NPs can be easily observed. The low magnification FESEM pictures are shown in Fig. 4a at 20 μm, and high magnification images of the products are shown in Fig. 4b at 1 μm^35,36^.

### EDX analysis

**Figure 5:**
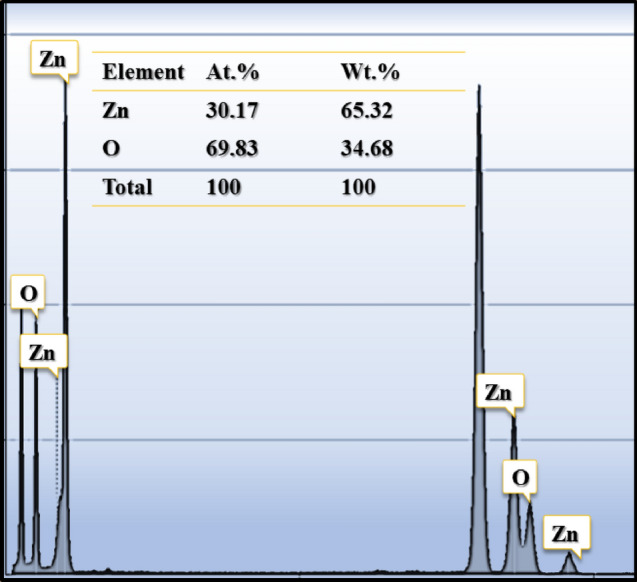
Edx spectrum of Chemical synthesized PVP-ZnO NPs.

The composition of PVP-ZnO NPs is investigated using Energy Dispersive Spectroscopy (EDS), as shown in Fig. 5. According to the EDS analysis, the synthetic compounds are made of zinc (Zn) and oxygen (O). No further peaks in the EDS spectrum connected to any contaminant are visible up to the instrument's detection limit. This demonstrates that the synthesized products are pure ZnO made of zinc (Zn) and oxygen (O)^37^.

### HRTEM analysis

**Figure 6:**
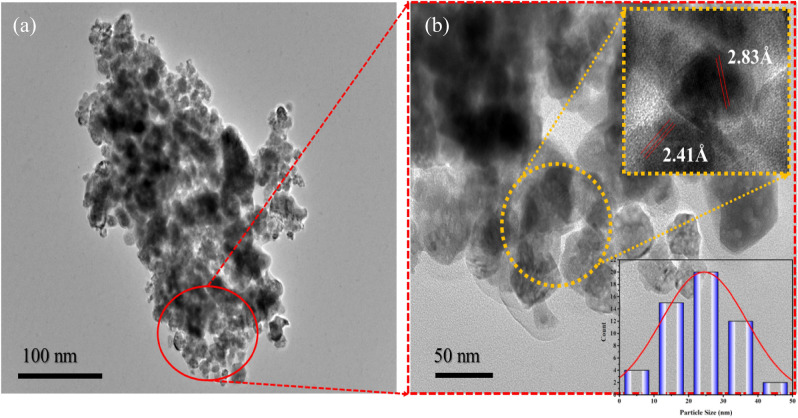
HRTEM images of PVP-ZnO NPs at different scale bars with size distribution histogram and lattice fringes (inset).

The HRTEM images of the created zinc oxide nanoparticles are shown in Fig. 6. The size of the produced ZnO-NPs was determined to be varying from 20 to 30 nm, which is in complete agreement with the value obtained from the XRD data, and the HRTEM images show that they are polycrystalline with a spherical structure. The higher-resolution HRTEM images also show that NPs are separated by a uniform interparticle distance rather than being in direct touch. The high-resolution transmission electron microscopy (HRTEM) image (Fig. 6b) provides clear evidence of the presence of the “002” plane in the wurtzite crystal structure of ZnO. The observed pattern consists of well-resolved and continuous fringes, with a measured lattice spacing of 2.83, confirming the characteristic arrangement of atoms within the ZnO crystal lattice.

### XPS analysis

**Figure 7:**
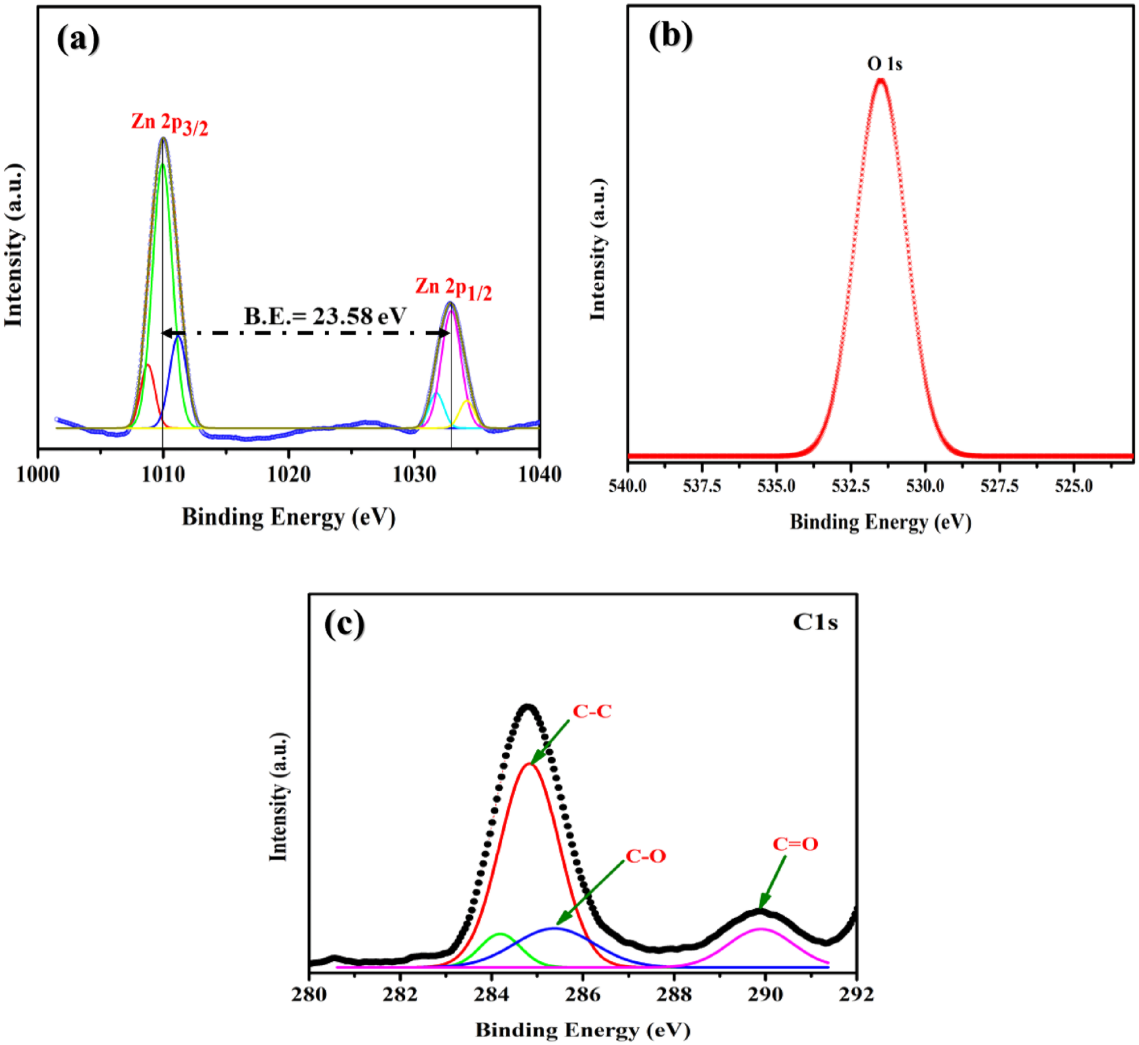
X-ray photoelectron images of synthesized PVP-ZnO NPs (**a**) Zn (2p_3/2_, 2p_1/2_), (**b**) O-1s state, and (**c**) C1s state.

XPS was used to analyze the elemental composition of ZnO nanoparticles and their states on the surface. As seen in Fig. 7, all of the peaks belong to the elements zinc and oxygen, which is consistent with the findings of the EDS. The binding energy peaks of Zn 2p_1/2_ and Zn 2p_3/2_ are found at 1033.6 and 1010.02 eV, respectively, in the Zn 2p spectrum (Fig. 7a). The fact that there is a difference of 23.58 eV in energy between the two peaks indicates that the valence state of the Zn element is + 2 and that it exists in the form of ZnO. According to the findings from the O 1s spectra (shown in Fig. 4b), the binding energy peak of the O 1s molecule displays symmetry, with 531.87 eV dissolving into a lattice ion. It has been demonstrated that ionic substitution occurs, producing oxygen substitution inside the lattice. The C1s are depicted in Fig. 7c. The BE distance between the two peaks in the C1s XPS spectrum of ZnO is 4.1 eV. The ZnO C1s XPS spectrum’s primary peak is 284.9 eV, and the satellite peak is 288.1 eV. The peak at 284.6 eV is ascribed to carbon contamination. The peak at 285.7 eV is associated with the C–O band. The peak at 288.8 eV is caused by surface carbonate species that are loosely bound, such as C=O, and carbon may be incorporated into the interstitial positions of the ZnO lattice.

### Photoluminescence study

**Figure 8:**
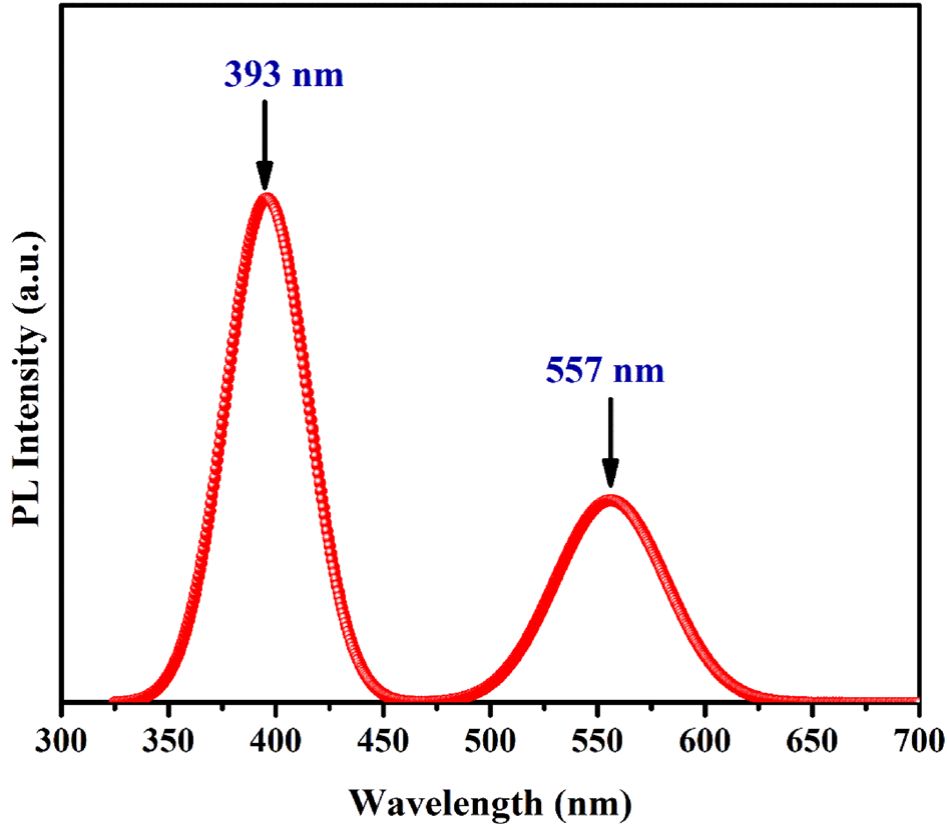
Photoluminescence emission spectra of ZnO.

To investigate the charge transfer and migration properties of PVP-ZnO NPs, the photoluminescence spectra (PL) were obtained. The PL emission intensity results from recombining photoexcited electron-/hole pairs; consequently, lower and higher PL emission intensities indicate lower and greater recombination of photogenerated charge carriers, respectively. The PL emission spectra of all synthesized photocatalysts are shown in Fig. 8. The obtained spectra can be divided into two distinct regions, one known as the band edge emission region, with a wavelength range of 375 to 395 nm. The recombination of conduction band electrons and valence band holes causes this first region of the emission spectrum. The second emission region in the wavelength range of 525 to 575 nm may be attributed to the defects emission region, where defects in the ZnO structure cause emission.

## Antibacterial activity

In the present research, the NPs diffusion technique was used to investigate whether or not PVP-ZnO NPs are effective against *E. coli* and *B. subtilis*. *E. coli* is a gram-negative bacterium, and *B. subtilis* is a gram-positive bacterium. This test covered both types of bacteria, which are representative of other bacterial strains basis on this feature. Also, according to the Centres for disease control and Prevention, *E. coli* is found in the environment, food, and untreated water. Hence, evaluation against it also means we can tackle other bacteria dwelling in the same sources. Because of their reduced toxicity, zinc oxide nanoparticles (PVP-ZnO NPs) were the subject of research to see whether or not they were useful as a possible raw material for various biological applications, most notably as an antibacterial agent.

**Figure 9:**
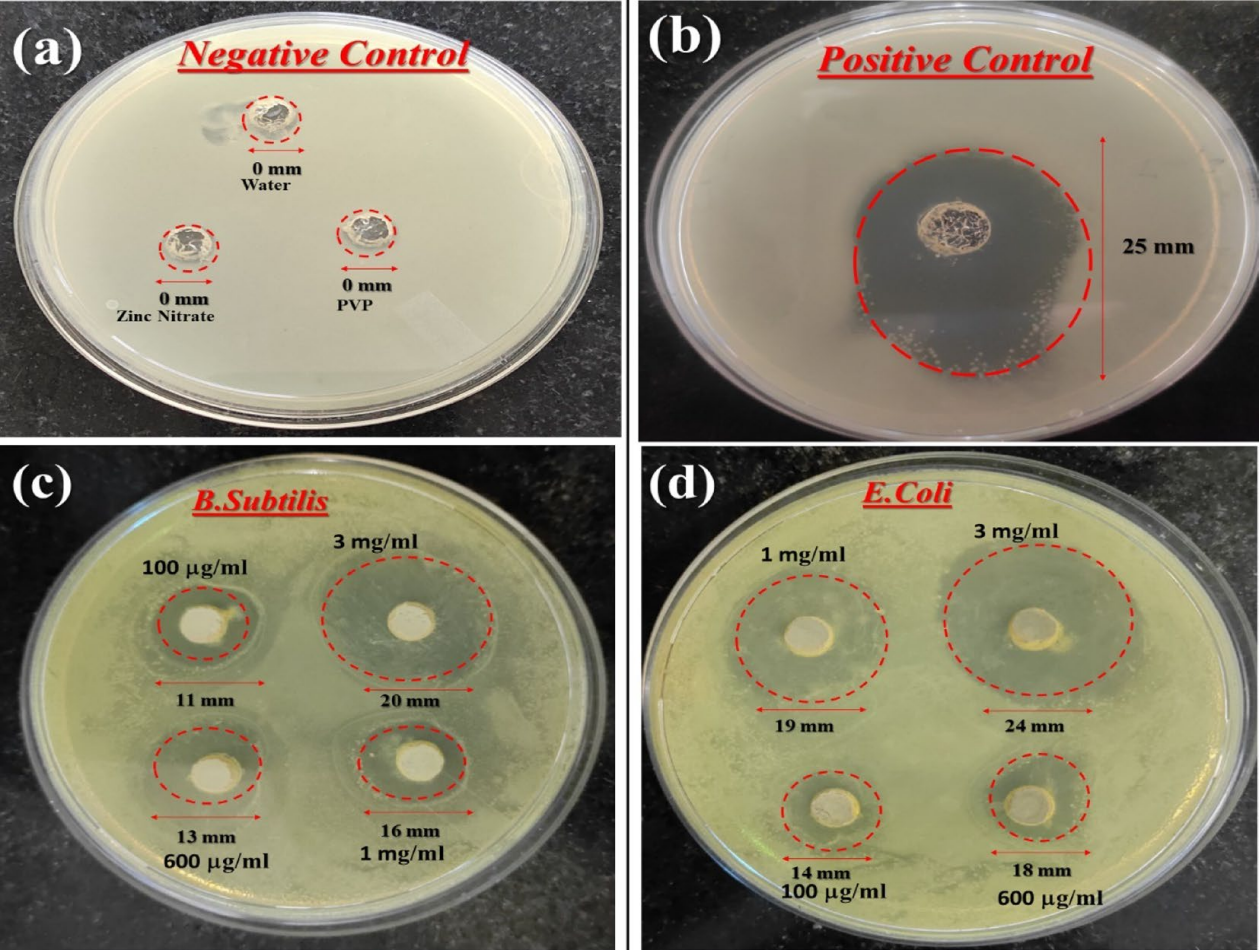
Agar plates with PVP-ZnO NPs that have zones of inhibition against bacteria (**a**) negative control, (**b**) positive control, (**c**) *E. coli*, and (**d**) *B. subtilis.*

**Figure 10:**
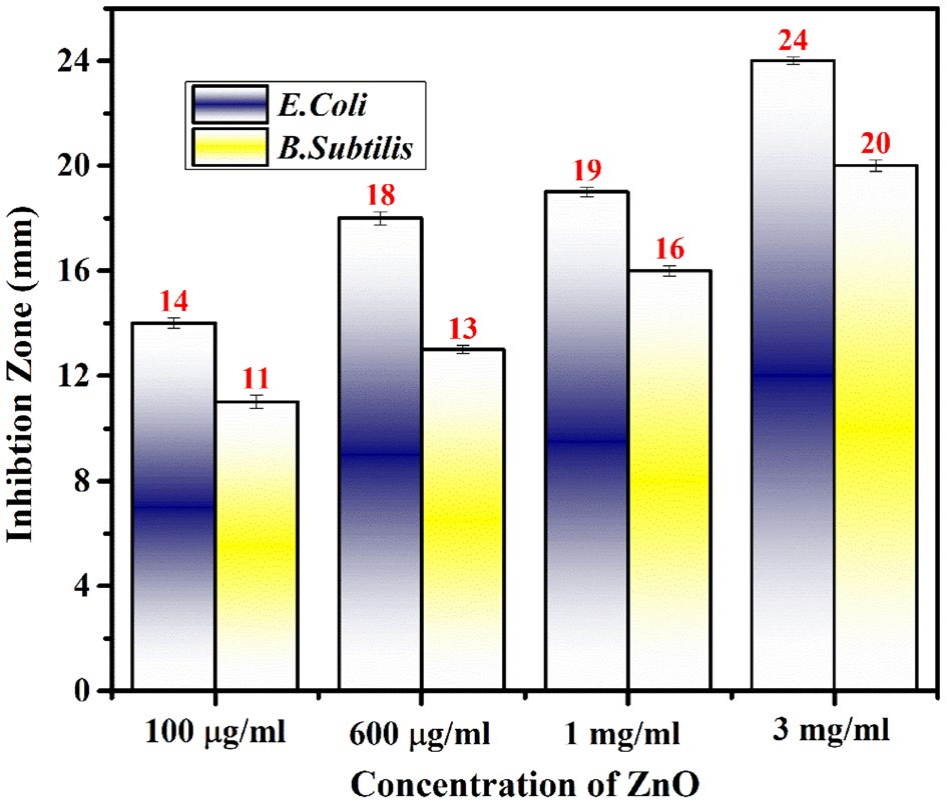
Histogram shows the zone of clearance by PVP-ZnO NPs.

Figure 9 depicts the dose-dependent enhancement of antibacterial activity, establishing a correlation between the increased concentration of ZnO nanoparticles (NPs) and improved activity. Our study findings reveal the potent antibacterial impact of PVP-ZnO NPs on *E. coli*, whereas their antibacterial effect on *B. subtilis* is comparatively modest. The disparity in growth inhibition between the two bacterial types can be attributed to the structural differences in their cell walls. *E. coli*, a gram-negative bacterium with a thin cell wall, is more susceptible to the antibacterial properties of PVP-ZnO NPs, whereas *B. subtilis*, a gram-positive bacterium with a thick cell wall, exhibits reduced sensitivity. In negative control, checked against water, zinc nitrate and PVP, no zone of inhibition is observed; in positive control, a clear zone of inhibition (25 mm) is observed. PVP-ZnO NPs were given to the subjects at 100 μg/mL, 600 μg/mL, 1 mg/mL, and 3 mg/mL, respectively. PVP-ZnO NPs had a maximal zone of inhibition of 20 mm ± 0.15 mm for *B. subtilis*and 24 mm ± 0.19 mm, for *E. coli*. Consequently, the larger surface area of ZnO nanoparticles is attributed to their increased antibacterial activity (Fig. 10).

PVP-ZnO NPs were specifically examined for their potential application as an antibacterial agent owing to their comparatively lower toxicity. The enhanced antibacterial effectiveness of PVP-ZnO NPs can be attributed to their increased surface area, oxygen vacancies, release of Zn2+ ions, and ability to disperse as reactive molecules. The increased specific surface area to volume ratio and reduced crystallite size of PVP-ZnO NPs lead to extra vacancies and reactive oxygen species (ROS) production. PVP-ZnO NPs exhibit antibacterial activity primarily due to the presence of oxygen vacancies influenced by Zn^2+^ ions. These vacancies generate reactive oxygen species (ROS) like hydrogen peroxide (H_2_O_2_), O^2−^, and OH, which can cause DNA damage and apoptosis^38,39^. The degree of contact between the nanomaterial and the bacteria cell membrane determines the shape-based antibacterial action. Spherical-shaped NPs have a large surface area exposed that can bind to the bacteria^40^. Table 5 shows the comparison detail of antibacterial study performance.

**Table 5:** Comparison detail of antibacterial study.

S.no.	Sample (nanoparticles)	Results	Mechanism	References
1.	ZnO	Enhanced antibacterial property	The high rate of production of Reactive Oxygen Species	^41^
2.	Cd-ZnO	Antibacterial action against a wide range of pathogenic organisms	The surface of CdO-ZnO nanocomposites produced hydrogen peroxide (H_2_O_2_), which discharged the positive charge of Cd^2+^	^42^
3.	ZnO	effective bacterial elimination	Promoted hydroxyl radical production	^43^
4.	ZnO/TiO_2_	*E. coli*-specific antibacterial activity	Interaction with highly reactive oxygen species that damaged the outer membrane or the inside of the bacteria	^44^
5.	ZnO	Enhanced antibactericidal action against *E. coli* and *B. subtilis*	ROS generation by nanoparticles that disrupt the functioning of bacterial cell	Present work

**Figure 11:**
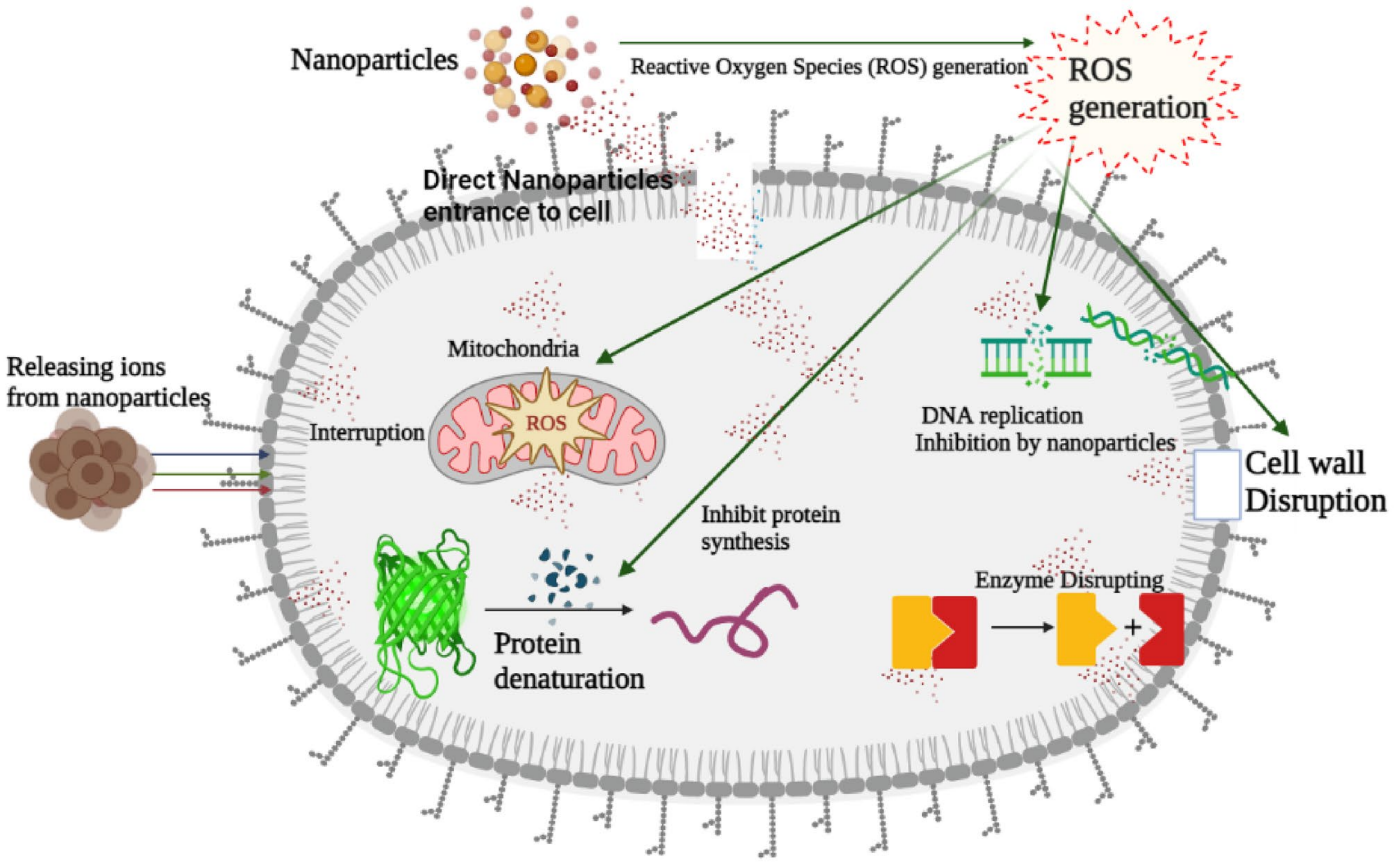
Mechanism of antibacterial.

The electrostatic interaction between positively charged Zn^2+^ ions from ZnO nanoparticles (NPs) and the negatively charged bacterial cell membrane results in transmembrane pores and disrupting membrane permeability. This is a feasible antibacterial activity of PVP-ZnO NPs, as seen in Fig. 11.

### Activities of the synthesised ZnO in scavenging free radicals

**Figure 12:**
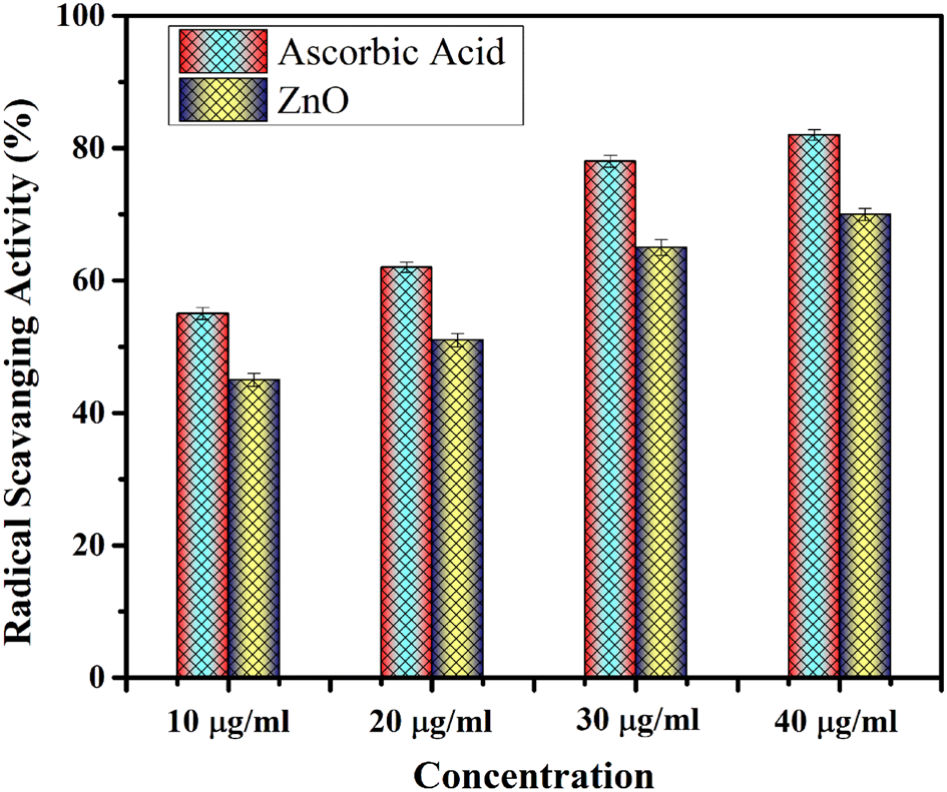
DPPH scavanging ability of PVP-ZnO NPs.

Free radical scavenging activity of synthesised PVP-ZnO NPs may be quickly and easily estimated using DPPH. PVP-ZnO NPs cause a drop in absorbance at 517 nm, confirming the color change from purple to yellow. By donating electrons from the oxygen atoms to the odd electrons of the nitrogen atoms, DPPH is converted into DPPH free radicals, and the molecule DPPH is formed. Surface oxidation–reduction processes occur when ZnO nanostructures are stimulated at the right wavelength, generating holes. ZnO, a semiconductor, was a trap for the electrons, interacting with oxygen and water to produce HO_2_. Organic dyes degraded because hydroxyl surface groups blocked the holes, releasing OH radicals. The antioxidant activity of ZnO NP’s was found in Fig. 12 to increase with the increase in concentration from 10 to 40 µg/mL.

## Photocatalytic study

**Figure 13:**
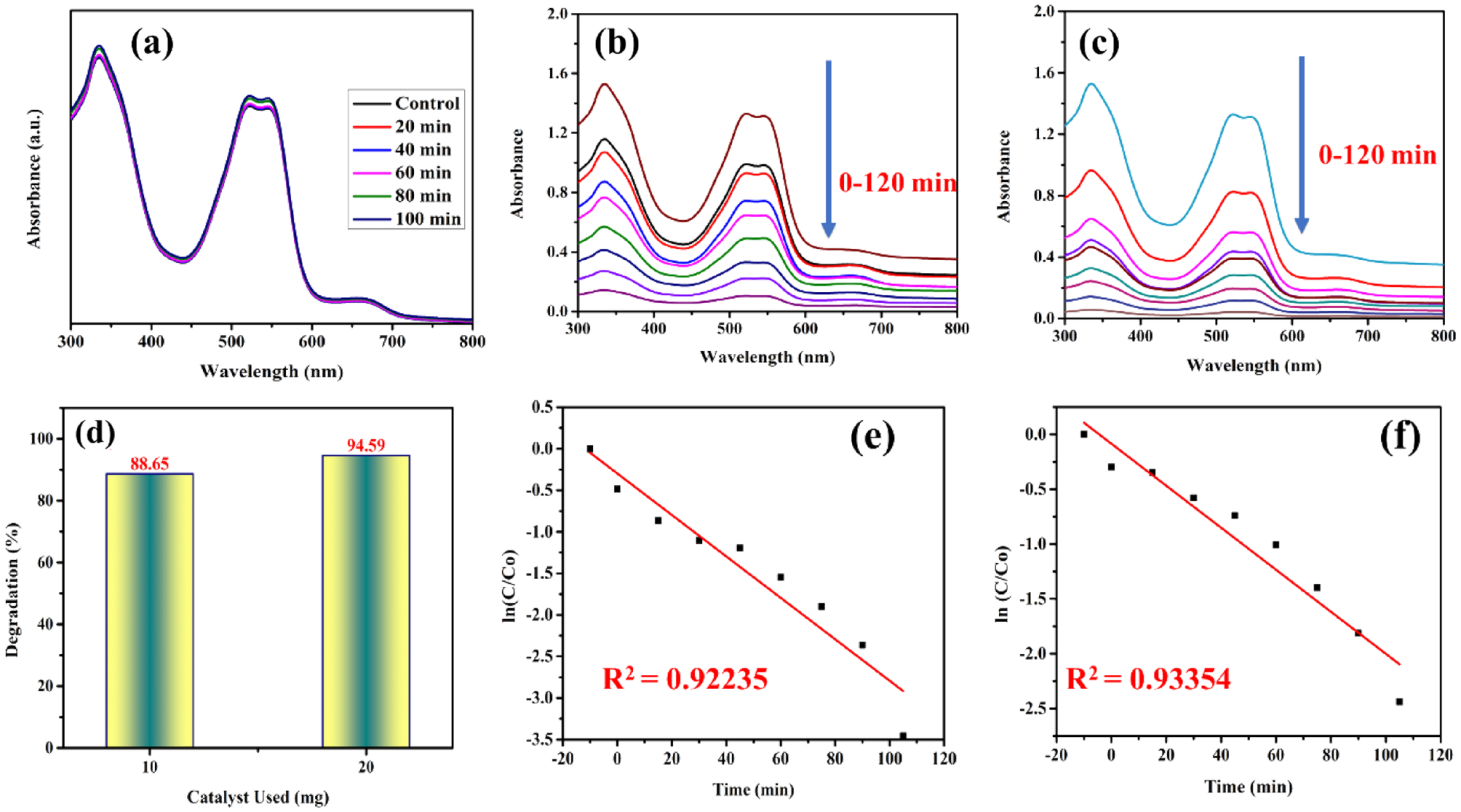
Shows (**a**) absorption spectra without photocatalyst, (**b**) absorption spectra of RR-141 with 10 mg catalyst, (**c**) absorption spectra of RR-141 with 20 mg catalyst, (**d**) degradation efficiency, (**e**) Pseudo-first-order kinetic (10 mg catalyst), and (**f**) Pseudo first-order kinetic (20 mg catalyst).

One of the most important uses of eco-friendly photocatalysts is the photodegradation of organic dye, namely reactive red-141 azo dye from the textile industry. To comprehend the possible uses of ZnO nanostructures created using a prepared PVP capping agent. The photocatalytic performance of a ZnO sample was evaluated by monitoring the degradation of reactive red (RR-141) dye (initial concentration: 5 mg/L) under direct solar light exposure over a defined duration. Absorption spectra of RR-141 dye solution with PVP-ZnO NPs for up to 120 min are depicted in Fig. 13a,c, respectively. Under the influence of solar light and in the presence of ZnO photocatalyst, the RR-141 azo dye’s highest absorption peak at 546 nm steadily reduced during the experiment as time passed, as shown in Fig. 13b,c. After 120 min, the absorption peak of RR-141 azo dye at 546 nm completely disappeared. This demonstrates the photocatalytic dye degradation potency of obtained PVP-ZnO NPs (88% with 10 mg catalyst and nearly 95% with 20 mg catalyst), as shown in Fig. 13d. Different shapes and sizes of nanoparticles influence the rate of photocatalysis as it changes the proportion of exposed polar faces. Because of the positive charge and more proportion of the ZnO facet, OH^−^ ions could preferentially adsorb on this face. This would result in a faster rate of formation of OH radicals and so dye degradation during photocatalysis^45^.

The rate of process degradation can serve as a measure for assessing the photocatalytic activity of the obtained PVP-ZnO NPs^46,47^. The modified Langmuir–Hinshelwood (L–H) equations are employed to account for solid–liquid interface reactions, allowing for a rationalized model. This model enables the characterization of steady-state photocatalytic reactions within a heterogeneous photocatalyst system. The pseudo-first-order reaction kinetics of PVP-ZnO NPs for the degradation of RR-141 azo dye has been estimated through the following equation:

$${\text{ln}}Co/C={k}_{1}t,$$

where k_1_ denotes the reaction rate constant for a pseudo-first-order photodegradation, A representation of ln (C0/C) vs. the irradiation duration for the RR-141 degradation catalyzed by each capped ZnO nanostructure is shown in Fig. 13e,f. This shows that pseudo-first-order kinetics governs the photodegradation of the azo dye RR-141 by ZnO photocatalysts. The resulting equation was used to determine the linear fit for each analysis. The slope provides the constant rate of change or k1. A pseudo-first-order kinetic equation governs the photocatalytic degradation reaction of an azo dye, according to the high R^2^ value (> 0.9).

**Figure 14:**
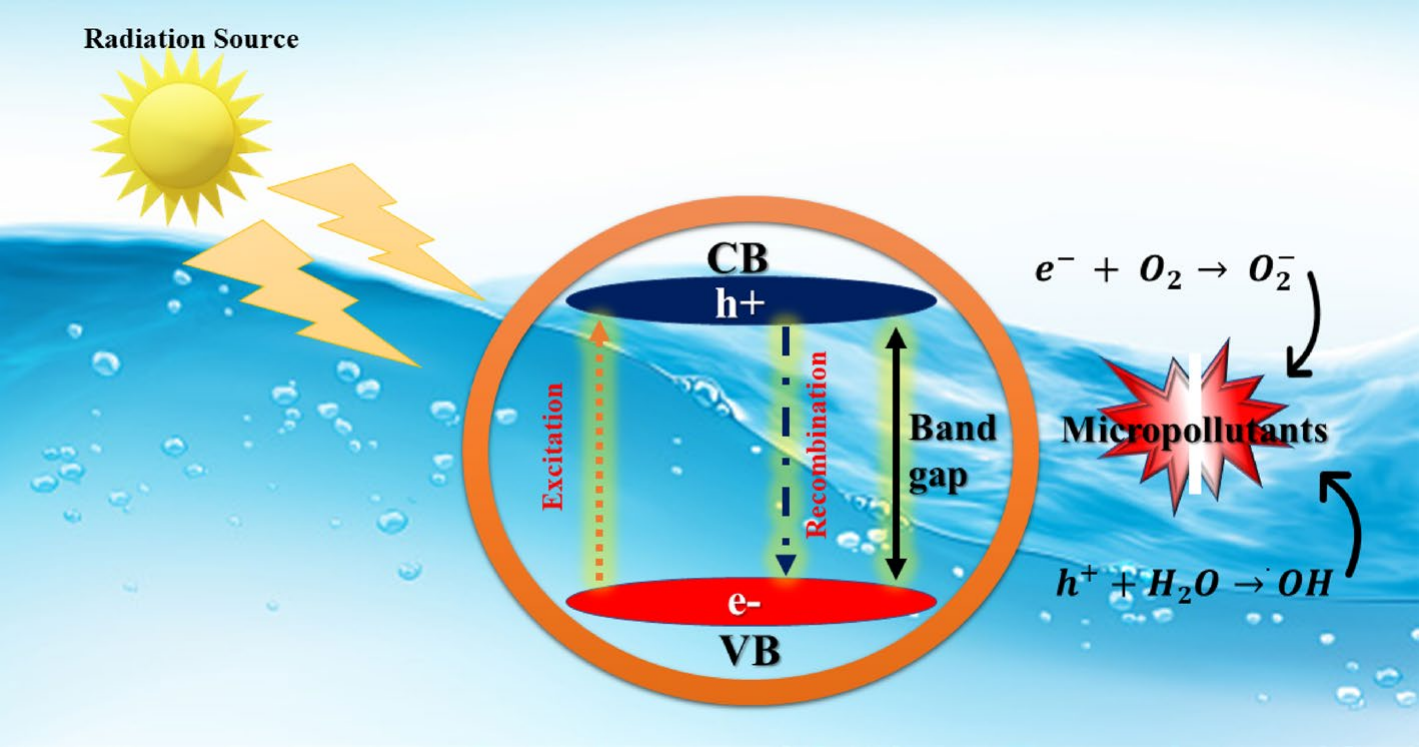
Photocatalytic mechanism.

Figure 14 depicts a schematic representation of the photocatalytic reaction mechanism.

When the energy of direct sunlight in terms of photons is equal to or greater than the bandgap of ZnO, the electrons receive energy, and electrons are transferred from the valence band to the conduction band, resulting in the formation of a hole (h^+^) and an electron (e^−^) in the valence band^48,49^. The pores react with water to produce. OH radicals, which are capable of oxidizing the organic pollutants^50,51^.

During the reduction process, the conduction band electron reacts with oxygen to produce^**.**^OH radicals. These radicals inhibit the production of organic contaminants. Under direct sunlight irradiation, these oxidation and reduction processes could degrade the organic pollutants. Based on earlier studies^53^, the following equation explains the photocatalytic reaction mechanism: The detailed summary of photocatalytic degradation of pollutants is shown in Table 6

**Table 6:** Summary of photocatalytic degradation of pollutants.

Sr. no.	Photo-catalyst (year)	Pollutant	Method of synthesis	Wave-length λ (nm)	Time (h)	Deg. (%)	Ref.
1	ZnO (2022)	Methylene Blue	Chemical	663	1.5	93.02	^52^
2	ZnO (2022)	Methyl Red	Chemical	492	2.5	93.19	^53^
3	ZnO⋅SiO_2_ (2021)	Methyl Orange	Chemical	465	1.5	91.02	^54^
4	Cu–ZnO (2019)	Direct Blue 15	Chemical	663	1	70.00	^55^
5	C–ZnO (2020)	Malachite green dye	Chemical	254	4.0	86.00	^56^
6	Au–ZnO	RhB	Green	553	3	97.20	^57^
7	ZnO	Reactive red 141	Chemical	546	2	89	^58^
8	ZnO	Reactive Red 141	Chemical	546	1.5	88	^59^
9	ZnO	Reactive Red 141	Chemical	546	4	94	^60^
7	ZnO	Methylene orange	Chemical	465	2	61	^61^
9	ZnO	Reactive Red-141	Chemical	546	2	94.59	Present work

5 $${\text{ZnO}}+Direct sunlight\to {\text{ZnO}} \left({e}_{cb}+{h}_{vb}\right),$$

6 $${\text{ZnO}}{(h}_{vb}^{+})+{\text{H}}{\text{O}}_{2}\to {\text{ZnO}}+{\text{H}}^{+}+{\text{OH}},$$

7 $${\text{ZnO}}{(h}_{vb}^{+})+{\text{OH}}^{-}\to {\text{ZnO}}+{\text{OH}},$$

8 $${\text{ZnO}}\left({e}_{cb}^{-}\right)+{\text{O}}_{2}\to {\text{ZnO}}+{\text{O}}_{2}^{-},$$

9 $${\text{O}}_{2}^{-}+{\text{H}}^{+}\to {\text{H}}{\text{O}}_{2},$$

10 $${\text{H}}{\text{O}}_{2}+{\text{H}}{\text{O}}_{2}\to {\text{H}}_{2}{\text{O}}_{2}+{\text{O}}_{2},$$

11 $${\text{H}}_{2}{\text{O}}_{2}+{\text{O}}_{2}^{-}\to {\text{OH}}+{\text{OH}}^{-}+{\text{O}}_{2},$$

12 $$Dye+{\text{OH}}^{.}\to Degraded Product.$$

## Conclusions

In this work, PVP-ZnO NPs have been successfully prepared via the co-precipitation method using PVP as a capping agent. The structural morphology, size, and configuration of PVP-ZnO NPs were studied using FTIR, XRD, FESEM, HRTEM, XPS and UV–Visible spectroscopic analysis, which indicates the formation of spherical-shaped ZnO NPs having a size varying from 20 to 30 nm. Further, the synthesized nanoparticles were evaluated for their antibacterial and photocatalytic potential. This study reveals the potent antibacterial impact of PVP-ZnO NPs against *E. coli* and *B. subtilis*. The antibacterial effect on *E. coli* is higher as compared to *B. subtilis*. This study also demonstrated the photocatalytic dye (reactive red-141) degradation potency of obtained PVP-ZnO NPs and degraded 88% and 95% using 10 mg catalyst and 20 mg catalyst, respectively. ROS species’ generation is the main factor for photocatalytic and antimicrobial studies, which was investigated through DPPH antioxidant assays. Thus, this study explores the effective platform for environmental remediation applications and delves into a way for future researchers to work on the multidisciplinary applications of metal oxide nanoparticles.

## Data Availability

The datasets used and analyzed during the current study are available from the corresponding author upon reasonable request.
